# Food insecurity and age at menarche among adolescent girls in Jimma Zone Southwest Ethiopia: a longitudinal study

**DOI:** 10.1186/1477-7827-9-125

**Published:** 2011-09-13

**Authors:** Tefera Belachew, Craig Hadley, David Lindstrom, Yehenew Getachew, Luc Duchateau, Patrick Kolsteren

**Affiliations:** 1 Department of Population and Family Health, College of Public Health and Medical Sciences, Jimma University, PO.Box 1104, Jimma, Ethiopia; 2 Department of Food Safety and Food Quality, Faculty of Bioscience Engineering, Ghent University, Coupure Links 653, B-9000 Gent, Belgium; 3 Department of Anthropology, Emory University, 207 Anthropology Building 1557 Dickey Drive, USA; 4 Department of Sociology, Brown University, Box 1916, Providence, RI 02912, USA; 5 Yehenew Getachew, Collage of Agriculture and Veterinary Medicine, Jimma University, Jimma, Ethiopia; 6 Department of Physiology & Biometrics, Faculty of Veterinary Medicine, Ghent University, Belgium; 7 Nutrition and Child Health Unit, Department of Public Health, Institute of Tropical Medicine, Nationalestraat 155, 2000 Antwerpen, Belgium

## Abstract

**Background:**

Age at menarche is the reflection of cumulative pre-adolescent exposure of girls to either adverse environment such as food insecurity or affluent living conditions. Food insecurity could result in inadequate nutrient intake and stress, both of which are hypothesized to have opposing effects on the timing of menarche through divergent pathways. It is not known whether food insecure girls have delayed menarche or early menarche compared with their food secure peers. In this study we test the competing hypothesis of the relationship between food insecurity and age at menarche among adolescent girls in the Southwest Ethiopia.

**Methods:**

We report on 900 girls who were investigated in the first two rounds of the five year longitudinal survey. The semi-parametric frailty model was fitted to determine the effect of adolescent food insecurity on time to menarche after adjusting for socio-demographic and economic variables.

**Results:**

Food insecure girls have menarche one year later than their food secure peer (median age of 15 years vs 14 years). The hazard of menarche showed a significant decline (P = 0.019) as severity of food insecurity level increased, the hazard ratio (HR) for mild food insecurity and moderate/severe food insecurity were 0.936 and 0.496, respectively compared to food secure girls. Stunted girls had menarche nearly one year later than their non-stunted peers (HR = 0.551, P < 0.001).

**Conclusion:**

Food insecurity is associated with delay of age at menarche by one year among girls in the study area. Stunted girls had menarche one year later than their non-stunted peers. Age at menarche reflects the development of girls including the timing of sexual maturation, nutritional status and trajectory of growth during the pre-pubertal periods. The findings reflect the consequence of chronic food insecurity on the development and well-being of girls in the study area.

## Background

Adolescence is a period of rapid transition to adulthood marked by biological changes including sexual maturation. Puberty is a period during adolescence characterized by transformation from a stage of reproductive immaturity to a stage of full reproductive competence. The sign of puberty in girls is menarche (the first menstruation) which occurs at younger age in high income countries [1-5] compared with developing countries in Africa [6-10] with minimal decline in those in Latin America [11-13] and Asia [14]. These disparities in age at menarche are related to the improvements in childhood nutrition and health in high income countries among other environmental factors [13,15,16].

There are different theories for the pathways through which environmental factors could influence of age at menarche. An evolutionary theory proposes that adverse childhood experiences [stress, anxiety, insecurity, food insecurity and poor care] accelerate the timing of menarche as an alternative reproductive strategy to maximize the chance of leaving descendents [17,18]. Studies have shown that girls who are exposed to adverse life events during the pre-pubertal periods have menarche at a younger age and resume fertility earlier than girls not exposed to such life events [18,19].

Unlike the above theory, the energetics theory suggests energy availability during childhood influences the timing of menarche [20]. It hypothesizes that girls who were exposed to a chronically food and nutrient constrained environment will track their growth more slowly, experience later pubertal development including menarche relative to their genetic potential and reach relatively small adult size compared with those children who were exposed to better food availability.

Adolescents in developing countries are exposed to adverse environmental conditions including food insecurity which could impact on their development and wellbeing. The pathways through which food insecurity can impact on time to menarche may either be through stress, anxiety and insecurity related to concerns around food availability at home [21,22] or through insufficient access to energy and other nutrients for normal health, growth and development [23-32].

It is not known whether food insecurity as an adverse condition would lead to either delayed menarche or early menarche given that it could result in both decreased access to food and increased stress and feeling of insecurity. Evidences from developing countries show that menarche occurs at an older age suggesting indirectly that the effect of food insecurity on age at menarche may be more through nutritional mechanisms [6,8-10,13]. Although food insecurity is a common in Ethiopia in general [33,34] and among adolescents in the study area in particular [28], there is no study that documented the relationship between girls' own experience of food insecurity and age at menarche. We previously reported how girls suffered from food insecurity in an Ethiopian context and the health consequences of that food insecurity has on girls [28,29]. We now document the effect of food insecurity on age at menarche. In this study, we test the competing hypotheses about the relationship between food insecurity and age at menarche among Ethiopian adolescents.

## Methods

### Study sample

Data for this report is obtained from a longitudinal family survey of youth in the context where a 5 years study is tracking the life events of adolescents as they transit to adulthood in Jimma zone southwest Ethiopia. The survey began in 2005 and involved adolescents and households from a total of 18 "kebeles" (villages) selected from Jimma city and three rural districts, namely Kersa, Dedo and Manna. Manna district is a coffee growing area, Dedo is highland and a vegetable growing area, while Kersa is an edible crop growing plain area with average altitudes of 1911, 2300, 1795 meters above sea level, respectively.

A census was done to generate list of all households which gave a sampling frame for random selection of 3,700 households from the total of 5,795 households in the list. A two-stage sampling plan was used to select the target sample of adolescents. Households were classified into urban (Jimma City), semi-urban (Serbo, Dedo and Yebbu Towns) and six rural communities (two in the vicinity of each of the three small towns). At the first stage, households were randomly sampled with the sample size in each "kebele" determined by the relative proportion of the study population in the "kebele" and the overall target sample size. In the second stage, one adolescent (a boy or a girl) was randomly selected from each household using a Kish Table [35]. Using this sampling strategy a total of 1059 boys and 1025 girls were interviewed in round one. This paper reports on female adolescents interviewed in both the first and the second rounds of the five year longitudinal family survey of youth (n = 900).

### Measurements

Structured household and adolescent level questionnaires were used to collect data. The questionnaires were interviewer-administered and translated in to Amharic and Oromifa languages and checked for consistency by other persons who speak both these languages and English. The household questionnaire included a household registry that collected socio-demographic information on all current resident and non-resident household members including information on their income and food security status. The heads of households responded to the household questionnaire. The adolescent questionnaire focused on issues related to adolescents' experiences of food insecurity, education, health and anthropometric measurements. The interview was conducted in a private place by an interviewer of the same sex as the adolescent respondent after the household interview was completed.

Adolescent food insecurity was measured using a four item index adopted from household food security questionnaires used in developing countries [23-26] by modifying the items that could be used at an individual adolescent level. The details of the methods are described elsewhere [28,29]. To summarize briefly, adolescents were asked whether in the last three months they (1) had ever worried about having enough food, (2) had to reduce food intake because of shortages of food or money to buy food, (3) had to go without having eaten because of shortage of food or money to buy food and (4) had to ask outside the home for food because of shortage of food or money to buy food. The index of food insecurity is defined as the number of items with a positive answer. The index has high internal consistency (Cronbach's Alpha = 0.81).

All data on background characteristics and food insecurity were collected on round one (year 1) of the survey. In round two of the survey, which was conducted one year after the baseline data, female interviewers asked girls whether they had experienced their menarche and the age at which the event had happened using a Non-verbal Responses Card Method [36]. In this method, the interviewers asked questions using a questionnaire and the respondents answered using a laminated A4 size card by inserting a pencil through round holes which are next to the possible responses on the respondent side of the card. The interviewers registered the random numbers next to the responses on the interviewer side of the card. The codes were converted to the actual responses during analysis.

To determine the level of stunting, which is an indicator of chronic malnutrition, trained 12 grade complete data collectors measured height to the nearest 0.1 cm using a stadiometer (SECA, Hannover Germany). Height for age z-scores were calculated using WHO Anthro-Plus software [37] which was coded as stunted(less than -2) and non-stunted (-2 and above).

Domestic work index (Workload) was obtained by asking respondents on how many days they were engaged in a variety of tasks including caring for animals, working on farm activities, fetching water and fuel, washing clothes, cooking, engaging in childcare, pounding or grinding grain and engaging in heavy labor tasks during the last seven days before the survey. A principal component analysis was used to develop a workload index based on the range of activities that adolescents were involved in. The first factor was taken based on eigen values and standardized. Higher values correspond to higher workload.

### Statistical analysis

The data were analyzed using STATA 10. The classical way to model the relationship between time-to-event response variable such as time to menarche is based on the Cox Proportional Hazards (PH) model [38]. First, bivariate Cox proportional Hazards Models were fitted to evaluate the marginal effects of different covariates on time to menarche. For the multivariable Cox PH analyses, different covariates were entered into the model simultaneously and relevant interactions between the covariates were tested. Only variables that showed a significant association with age at menarche in the bivariate analyses were entered in the multivariate model. Since girls are nested (clustered) within villages, this clustering was taken into account by adding a gamma distributed random effect (frailty) to the Cox PH model [39]. Normality of the continuous variables was checked visually using Q-Q plots and household income was divided into tertiles as it is not normally distributed. We present the results as means, proportions and hazard ratios with 95% confidence intervals.

The study was ethically approved both by the Brown University IRB and by Jimma University Ethical Review Board before the beginning of the data collection.

## Results

After one year follow up a total of 900 girls were interviewed for age at menarche out of 1025 enrolled in the study in round one (125 girls were lost to follow up). Baseline characteristics of girls during the first round survey are presented by food security status in Table 1. The mean age of girls was 14.8 (sd = 1.3) years for food secure and 14.8(sd = 1.4) years for food insecure girls (P > 0.05). There was no significant difference in food insecurity by place of residence (P > 0.05). Food insecure girls had higher workload compared with food secure peers (P < 0.001) and larger proportion of food insecure girls were part of male headed households (P < 0.001).

**Table 1:** Characteristics of the girls at the first round survey (2005) by Food Security status.

Variables	Food Secure (n = 675)	Food Insecure (n = 225)	*P*
Mean age (± SD), Years*	14.8(1.3)	14.8(1.4)	*0.956*
Place			
Urban	36.3%	40.0%	*0.218*
Semi urban	28.0%	30.7%	
Rural	35.7%	29.3%	
Sex of the household head			
Male	78.7%	91.6%	*< 0.001*
Female	21.3%	8.4%	
Household income Tertile			
Low	34.8%	33.3%	*0.043*
Middle	30.2%	38.7%	
High	35.0%	28.0%	
Nutritional status			
Normal	77.2%	80.8%	*0.309*
Stunted	22.8%	19.2%	
Work index, mean(± SD)*	33.7(17.3)	40.1(17.5)	*< 0.001*
Mean highest grade completed(± SD)*	5.3(2.7)	5.2(2.6)	*0.014*

Source: Jimma Longitudinal Family Survey of Youth; Round 1, 2005-2006.

*An independent Samples T-test was used to compare means.

Chi-square test was used to compare portions.

Percentages are calculated out of column totals.

At the end of round two out of the 900 girls interviewed, 629 (69.9%) had experienced menarche. The median age at menarche for the whole sample was 14 years. The median age at menarche differed significantly between the different places of residences, with urban girls having menarche on average one year earlier than semi-urban and rural counter parts, the values being 14 years for the urban (range 13.88 to14.12 years), 15 years(range 14.91 to 15.09 years) for semi-urban areas and 15 years(range 14.99 to15.01 years) for rural areas. Similarly, food insecure adolescents had menarche one year later than those who were food secure, and stunted girls had their menarche one year later than their non-stunted peers.

**Figure 1:**
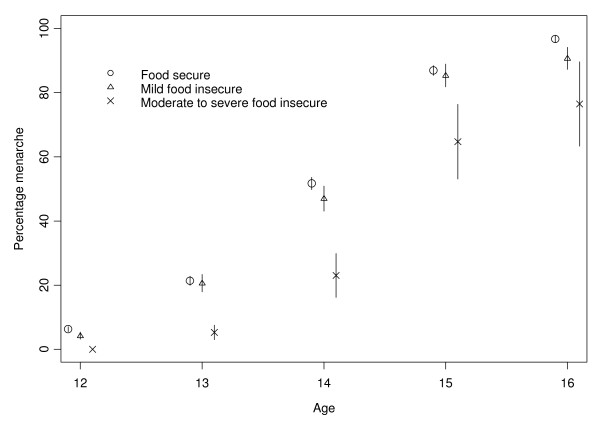
**Percentages of girls having experienced menarche as a function of age in years for the three different food insecurity groups**. The length of the bars indicates ± standard error.

At the of age of 14 years, for instance, only 23.0% of the girls with moderate/severe food insecurity had already experienced menarche, compared to 47.0% and 51.7% in the mild food insecurity and food secure groups, respectively (Figure 1).

The results of the bivariate Cox regression analyses are presented in Table 2. Food insecurity (P = 0.003), being a member of female headed household (P = 0.049) and place of residence (P < 0.05), being stunted (P < 0.001), workload (P = 0.022) and highest household income tertile (P = 0.016) had a significant association with time to menarche in the bivariate analysis. The hazard of having menarche among girls with mild food insecurity and moderate/severe food insecurity were 0.906 (95% CI: [0.743; 1.103]) and 0.417 (95% CI: [0.234; 0.745]), respectively compared to food secure ones. The hazard of having menarche among stunted girls was 0.546 (95% CI: [0.399; 0.747) compared to non-stunted girls (P < 0.001).

**Table 2:** Crude (bivariate analysis) from the Cox proportional Hazards Model with time to menarche as response variable.

Covariates	Crude HR (95% CI)	*P*
Adolescent Food insecurity		
Food secure	1.000	
Mild food insecurity	0.906 (0.743,1.103)	*0.326*
Moderate/Severe food insecurity	0.417 (0.234,0.745)	*0.003*
Place of Residence		
Jimma City	1.000	
Small Towns	0.817 (0.682,0.979)	*0.028*
Rural	0.759 (0.634,0.910)	*0.003*
Nutritional status(Height for age z-score)		
Normal	1.00	
Stunted	0.546 (0.399,0.747)	*< 0.001*
Work Index	0.994 (0.990,0.999)	*0.022*
Household income Tertiles		
Low	1. 000	
Middle	1.011 (0.834,1.126)	*0.911*
High	1.264 (1.045,1.529)	*0.016*
Head of the household		
Male	1.000	
Female	1.213 (1.001,1.469)	*0.049*

Source: Jimma Longitudinal Family Survey of Youth; Round 1, 2005-2006; Round 2, 2006-2007.

Data on predictors were collected one year before the dependent variable (age at menarche).

HR = Hazard Ratio.

CI = Confidence Interval.

Compared to urban girls, the hazard of having menarche among girls in a small towns was 0.817 (95% CI: [0.682; 0.979]), while the hazard of menarche among rural girls decreased further to 0.759 (95% CI: [0.634; 0.910]). Girls in the households with the highest income tertiles had the higher hazard of having menarche compared to those in the low income tertile (HR: 1.264 (95% CI: [1.045; 1.529]).

The hazard of having menarche among girls in female headed households was 1.213 (95% CI: [1.001; 1.469]) compared to girls in male headed households. Girls who were members of female headed households had menarche at earlier age compared to those in the male head households.

In the multivariable model, moderate/severe food insecurity had still a significant impact (P = 0.019) on time to menarche after adjusting for place of residence, household income, the sex of the household head and place of residence. The hazard of menarche showed a significant decline to 0.936 (95% CI: [0.756; 1.158]) and 0.496 (95% CI: [0.276; 0.892]), respectively for mild food insecurity and for moderate/severe food insecurity compared to food secure girls. Similarly, stunted girls had lower hazard of having menarche compared with their non-stunted peers (HR = 0.551, P < 0.001). Place of residence, household income, work index and sex of the household head did no longer have a significant impact on time to menarche on the multivariable analyses (Table 3).

**Table 3:** Adjusted hazard ratios (multivariate analysis) from the Cox Proportional Hazards model with time to menarche as response variable.

Covariates	Adjusted HR (95% CI)	*P*
Adolescent Food insecurity		
Food secure	1.000	
Mild food insecurity	0.936 (0.756,1.158)	*0.542*
Moderate/Severe food insecurity	0.496 (0.276,0.892)	*0.019*
Place of Residence		
Jimma City	1.000	
Small Towns	0.859 (0.705,1.045)	*0.128*
Rural	0.885 (0.951;1.126	
Nutritional status(Height for age z-score)		
Normal	1.000	
Stunted	0.551 (0.402;0.755)	*< 0.001*
Work Index	0.997 (0.992;1.003)	*0.383*
Household income Tertiles		
Low	1.000	
Middle	1.004 (0.818,1.233)	*0.968*
High	1.195 (0.967,1.476)	*0.098*
Head of the household		
Male	1.000	
Female	1.135 (0.927,1.389)	*0.220*

Source: Jimma Longitudinal Family Survey of Youth; Round 1, 2005-2006; Round 2, 2006-2007.

Data on predictors were collected one year before the dependent variable (age at menarche).

HR = Hazard Ratio.

CI = confidence interval.

## Discussion

Food insecurity is prevalent among adolescents in the study area [28,29]. Our results showed that food insecurity is associated with delayed age at menarche and food insecure girls had their menarche on average one year later than their food secure peers. These findings can be well explained by the energetics theory where delay in age at menarche among food insecure adolescents may be associated with inadequate nutrient intake [20]. In the situation of food insecurity individuals use food related coping strategies that would lead to decreases in the quantity and quality of food consumed [25,40,41]. Analysis of data from the same cohort of adolescent showed that food insecure girls have low quality diets [Unpublished]. Decreased food intake affects the maturation of the hormonal system that controls the occurrence of menarche. After birth, the hypothalamo-pituitary-gonadal axis continues to mature until menarche and the first years of menstruation. This process is characterized by changes in amplitude and pulsatility of Hypothalamic Gonadotrophine Releasing Hormon [42]. This development requires adequate supply of energy and other nutrients which are unlikely to be met in food insecure situation. The longstanding negative effects of childhood food insecurity on reproductive function of adult women has been also evidenced following the Dutch famine [43].

The results also showed that stunted girls had delayed age at menarche similar to the reports of studies in Senegal and Kenya which indicated that post-menarcheal girls had better nutritional status than pre-menarcheal ones [44,45]. Improved nutrition during early childhood has been reported to result in earlier fertility [5,16,46].

The mean age at menarche observed among girls in the study area (14 years) is higher than the age at menarche in developed countries [1,5,47] and even later than age at menarche for girls in developing countries in Africa [7-10], Asia [14,48] and Latin America [11,12].

Although menarche is mainly (50-70%) determined by genetic factors [49-51], environmental factors such as food insecurity have also a role in predicting age at menarche. Delayed age at menarche is associated with decreased fertility [52] poor reproductive function and other health problems including osteoporosis at later life [53], while early menarche is associated with breast cancer [54]. In the study area's context, the observed overall delay in age to menarche is indicative of the pre-pubertal suboptimal nutrition of girls in the study area who may have been suffering from the consequences of chronic food insecurity; which gives public health significance to the finding. These results highlight clues to the fact that the cumulative nutritional histories of girls in the study area may not be optimal during the pre-pubertal childhood periods. This will have intergenerational effects as childhood malnutrition leads to malnutrition during adulthood [55].

It was also observed that compared to girls in the urban areas, those in the rural and semi-urban areas had menarche one year later, which is consistent with the reports of other studies in developing countries [7-10] and in northern part of Ethiopia [56]. The difference could be due to the fact that larger proportion of girls in the urban areas were from households in the higher income tertile and rural girls have higher workload compared to those in the urban and semi urban areas. Both these factors increase the probability that urban girls have earlier menarche. However, the differences in age at menarche between urban and rural girls did not persist in the multivariable model.

Other reports indicated that vigorous physical activity is associated with decreased estrogen secretion and increased age at menarche due to the disturbance of GnRH pulsatility [57,58]. Although our data showed a significant association between work index and delayed age at menarche in the bivariate model, the association disappeared when adjusting for other covariates suggesting that the effect of workload may be mediated by other variables in the model.

It is possible that the effects of food insecurity could be mediated through stunting. However, our analysis showed that there is no correlation between food insecurity and stunting (P = 0.309) and the change in the effect of food insecurity when entering stunting into the model was not significant indicating that there is no mediation.

We used menarche as it is the most easily assessed and reliable self-reported aspect of female sexual maturity [59]. To reduce social desirability bias due to the private nature of the subject, we used a Non-verbal Response Card Method in interviewing girls about the occurrence of menarche and their age at the event [36]. The non-verbal Response Card Method is a local improvisation of the Computer Assisted Self Interview [CASI] method which is validated for use in developed countries [60,61].

In conclusion, food insecurity is associated with delay of age at menarche among girls in the study area. Stunted girls had menarche on average one year later than their non-stunted peers. Age at menarche reflects the development of girls including the timing of sexual maturation, nutritional status, and trajectory of growth during the pre-pubertal periods and wellbeing of girls. The fact that girls in the study area had menarche later than girls in developed countries and other low income countries reflects the consequence of chronic food insecurity on the development and well-being of girls in the southwest Ethiopia.

## Competing interests

The authors declare that they have no competing interests.

## Authors' contributions

The authors' responsibilities were as follows-- DL, CH, TB: designed and supervised the study and ensured quality of the data and made a substantial contribution to the local implementation of the study and PK, LD, YG, DL, TB, CH, and contributed in the analysis and interpretation of the data. TB, the corresponding author wrote the manuscript and had the final responsibility to submit it for publication. All authors read and approved the final manuscript.
